# Assessing the Durability of One-Shot Stimulus-Control Bindings

**DOI:** 10.5334/joc.218

**Published:** 2022-04-07

**Authors:** Peter S. Whitehead, Christina U. Pfeuffer, Tobias Egner

**Affiliations:** 1 Department of Psychology & Neuroscience, Duke University, Durham, NC 27708, USA; 2 Catholic University of Eichstätt-Ingolstadt, Department of Psychology, Ostenstraße 25, 85072 Eichstätt, Germany; 3 Center for Cognitive Neuroscience, Duke University, Durham, NC 27708, USA

**Keywords:** Cognitive Control, Action and perception, Learning

## Abstract

It has been proposed that cognitive control processes may be implemented in a contextually appropriate manner through the encoding, and cued retrieval, of associations between stimuli and the control processes that were active during their encoding, forming “stimulus-control bindings” as part of episodic event files. Prior work has found strong evidence for such a mechanism by observing behavioral effects of stimulus-control bindings based on a single pairing (one-shot learning). Here, we addressed the important question of how durable these one-shot stimulus-control bindings are. Over three experiments, we investigated the durability of one-shot stimulus-control bindings in relation to both the passage of time and the number of intervening events between the encoding (prime) and retrieval (probe) of the stimulus-control bindings. We found that stimulus-control bindings are quite robust to temporal decay, lasting at least up to 5 minutes in the absence of similar intervening events. By contrast, binding effects were more short-lived in the face of interference from the encoding of similar events between the prime and probe, with a maximum duration of ~2 minutes. Together, these results shed new light on the characteristics of the binding mechanisms underlying the integration of internal control processes in episodic event files and highlight that interference, rather than temporal decay, may be the main limiting factor on long-term effects of item-specific one-shot control learning.

## Introduction

*Cognitive control* describes a collection of processes underlying the ability to coordinate behavior in line with internal goals and the current state of the environment (Miller & Cohen, 2001). This requires the maintaining and shielding of currently goal-relevant stimuli and how they relate to actions – i.e. task-sets – as well as updating those task-sets in response to pertinent changes in one’s goals or circumstances (i.e., task switching; Monsell, 2003). Much research has been devoted to the critical question of how cognitive control processes are recruited and regulated so as to be applied in a context-appropriate manner (Blais et al., 2007; Botvinick et al., 2001; Verguts & Notebaert, 2008). One influential proposal is that control *settings* (or states) can become mnemonically associated with a particular context or stimulus, such that, for instance, a busy intersection on one’s daily commute can serve as a retrieval cue for an appropriate task-set and level of attentional focus (Abrahamse et al., 2016; Braem & Egner, 2018; Egner, 2014). However, many aspects of this hypothesized stimulus-control learning process remain poorly understood. One such unknown characteristic is the durability of mnemonic bindings between stimuli and control processes, which is the focus of the present study.

One specific way in which this stimulus-control binding process has been conceptualized is within the framework of *event-coding theory* (Hommel et al., 2001; Frings et al., 2020). Briefly, a long line of research suggests that the various features of an experienced event – such as a trial in a cognitive task – are bound together into an *event file* in episodic memory and, if any of those features reoccur later, this event file gets retrieved from memory to aid fast, event-appropriate processing. Event files have been shown to include the binding of different stimulus features (Treisman & Gelade, 1980), of stimuli with the actions performed in response to them (Hommel et al., 2001), and of task-specific semantic stimulus classification guiding those actions (e.g., stimulus classifications; Moutsopoulou et al., 2015; Pfeuffer et al., 2017). To account for observations of context-specific control binding, this framework has been extended via the proposal that internal states, such as the cognitive control processes active during a given event, are also encoded as part of the episodic event file (e.g., Egner, 2014). In turn, this facilitates fast retrieval of context-appropriate control settings when re-encountering the same situation in the future (Egner, 2014; Spapé & Hommel, 2008; Abrahamse et al., 2016). For instance, in the context of the current experiments (based on Whitehead et al., 2020), if a stimulus is encountered in the context of the need to switch task sets, control processes facilitating task switching (which we here refer to as *switch-readiness*) would become bound to the stimulus and retrieved when that stimulus is reencountered in the future.

Encoding such integrated event files and retrieving them when re-encountering the event features is beneficial in the kind of stable world in which human cognition has evolved. Contrary to laboratory settings, there the events of the past are often very similar to the events of the present, and similar stimuli or situations tend to require the same responses and/or cognitive processing strategies. Through systematic violations of this assumption of stability, however, we can experimentally create situations where the previously-required and currently-required cognitive settings (including cognitive control states) match versus mismatch. Comparing situations that conform to participants’ implicit expectations for stable requirements to those mismatching situations allows us to reveal event coding processes via the performance cost incurred in the latter compared to the former scenario.

Many studies on the contextually-appropriate implementation of cognitive control have interpreted their findings through the lens of this *episodic control binding hypothesis* (e.g., Dignath et al., 2019; Jiang et al., 2015; Spapé & Hommel, 2008), but the strongest empirical evidence to support this proposal derives from two recent studies demonstrating the encoding and retrieval of one-shot, item-specific stimulus-control bindings (Brosowsky & Crump, 2018; Whitehead et al., 2020). Specifically, these authors observed reduced task-switching costs (Whitehead et al., 2020, see also 2021) or flanker congruency effects (Brosowsky & Crump, 2018) for re-occurrences of a specific stimulus if that stimulus had previously been presented under a high versus low control demand – that is, during a task switch rather than a task repeat trial, or on an incongruent rather than a congruent trial. Notably, Whitehead and colleagues (2020) controlled for the repetition of the actual task set associated with a given stimulus, such that the observed reduction in switch cost must be driven by the retrieval of control processes involved in switching per se (switch-readiness) rather than the retrieval of a specific task set. The fact that these results were obtained in the context of a single prior stimulus-control state pairing (“one-shot learning”) supports the episodic binding hypothesis, because it rules out the possibility that the effect was mediated by incremental, non-episodic learning processes (like reinforcement learning, cf. Botvinick et al., 2001). Having established the basic existence of one-shot stimulus-control bindings, we here ask a critical next question: For how long are one-shot stimulus-control bindings effective in modulating behavior? Put another way, what is the durability of stimulus-control bindings formed via a single experience?

A growing body of literature has begun to directly investigate the durability of episodic event file bindings (Frings et al., 2015; Moutsopoulou & Waszak, 2013; Pfeuffer et al., 2017; see also Frings et al., 2020). For instance, it has been found that stimulus-classification bindings encoded during a prime phase (the initial presentation of a particular stimulus-classification ensemble) affected performance up to ~56 trials (~ 3 minutes) after encoding during a ensuing probe phase when the same stimulus was presented again (Pfeuffer et al., 2017). Moreover, not all bindings in an event file appear to be created equal. Stimulus-classification bindings have been found to survive for longer than stimulus-action bindings (about 27 minutes versus 3.7 minutes, respectively; Moutsopoulou & Waszak, 2013; Pfeuffer et al., 2017) and bindings between irrelevant task elements seem to only last up to one and a half minutes after encoding (Frings et al., 2015; see also Horner & Henson, 2009, 2011). However, also within this literature there is contrasting empirical evidence that such bindings might only last a few seconds after a one-shot presentation, as a function of the relevance of the features (e.g., Frings, 2011; Hommel & Colzato, 2004; Hommel & Frings, 2020; Moeller, Pfister, Kunde, & Frings, 2016). Taken together, this line of research suggests that the durability of event file bindings formed by a single exposure may be in the range of a few seconds to minutes, but also appears to be highly dependent on the specific binding type. Notably, however, no previous study has examined the durability of bindings between a stimulus and control settings of the type revealed in Whitehead et al. (2020).

Furthermore, prior research has probed the durability of event bindings almost exclusively as a function of the intervening number of trials between a prime and a probe trial, which does not allow one to explore the influence of two key factors affecting the maintenance of these bindings. Specifically, we propose that there are two factors that could limit the durability of event bindings, one being interference from subsequent encoding of new, similar episodic events, and the other being “passive” decay of the binding’s memory traces over time (Brown, 1958; Lewandowsky et al., 2009; see also Moeller & Frings, 2017). Though evidence for temporal decay of short-term episodic memories is mixed (Altmann, 2011; Mercer & McKeown, 2014; c.f. Oberauer & Lewandowsky, 2013), the extent to which bindings in episodic event files are susceptible to interference or temporal decay remains as of yet untested.

Therefore, in the present study, we assessed the durability of one-shot episodic stimulus-control bindings in two different ways. Specifically, we present three experiments that investigated both the pure temporal durability of one-shot stimulus-control bindings without interference from the encoding and retrieval of similar events, as well as their durability in the face of competing episodic event interference. We find that effects of one-shot stimulus-control bindings are robust over time in the absence of intervening events, with successful implementation of these stimulus-control bindings as long as 5 minutes after encoding. Further, retrieval effects are short-lived (~2 minutes) and dependent on task demands in the face of interference from the encoding of similar events. We address the qualifications and implications of these findings in the discussion.

## Experiment 1

In Experiment 1 we examined the durability of one-shot stimulus-control bindings in the face of interference from similar events being processed between the encoding and retrieval of the stimulus-control bindings. Here we used a modified version of the prime-probe task-switching design of Whitehead and colleagues (2020). There, each object image used in a cued task-switching protocol is presented once as a prime and, with a lag of several trials, once as a probe. The basic prediction of the episodic control binding hypothesis is reduced switch costs for probe items that were presented on a task *switch* versus *repeat* trial during prime exposure. In the current experiment, we modified this design via a manipulation of the temporal duration between the prime and probe stages (30 seconds to 7 minutes). During this time between prime and probe, participants completed a filler task – a task-switching task using trial-unique stimuli that did not repeat after initial presentation. This design allowed us to investigate the durability of one-shot stimulus-control bindings in the face of interference from the intervening creation of similar event files in episodic memory during the filler task.

### Method

#### Subjects

For .80 power with an α of .05 to detect an item-specific stimulus-control binding effect (i.e., a retrieval effect), using effect size estimates (β = |12.5|) based on previous work (Whitehead, et al., 2020; Brosowsky & Crump, 2018; Pfeuffer et al., 2017), we modeled a set of simulated mixed models using simr() which indicated we needed to run at least 250 participants. We thus chose to terminate data collection at 274 valid participants. In Experiment 1, we excluded 42 additional participants from the final analysis for performing at < 50% accuracy resulting in a final sample of N = 232 (mean age = 37.34 years, SD = 10.35; 107 Women, 124 Men, and 1 no response; 180 White). All participants in the current and following experiments were recruited from Amazon Mechanical Turk and provided informed consent in accordance with the policies of the Duke University Institutional Review Board. Participants were compensated at $9 per hour. Workers had to have a US-based IP address, more than 100 approved HITs, and greater than 95% acceptance rate to participate.

#### Procedure

**Figure 1:**
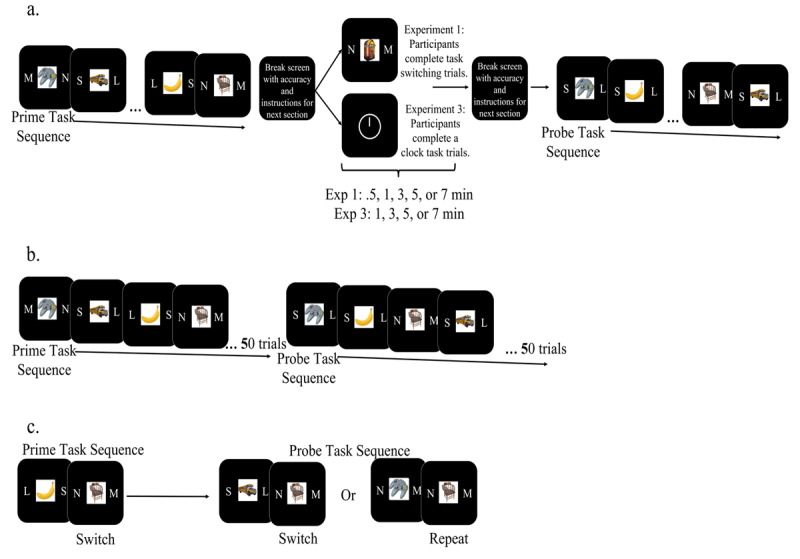
The paradigm **(a.)** for Experiments 1 and 3, illustrating the prime and probe phase separated by either a task-switching task (Exp. 1) or a clock task (Exp. 3) that lasted for .5 (Exp. 1 only), 1, 3, 5, or 7 minutes (Exp. 1 & 3). For each task-switching task trial in these experiments, the stimulus is presented in the center of the screen with letters on either side indicating the classification task and response mapping. The prime and probe phases lasted for 8 trials each, respectively. The paradigm **(b.)** for Experiment 2 involved the continuous presentation of task-switching trials for 100 trials in each block, with no intervening filler task between prime and probe phases. Illustration **(c.)** of the manipulation of probe trials as either task switch or repeat trials, while keeping response mapping and completed task constant between the prime and probe presentation of an item (here, the example being the chair).

The task (***Figure 1***) was a modified version of a basic cued task-switching protocol designed by Whitehead and colleagues (2020) where participants classified items according to either size (smaller or larger than a shoebox) or to whether the item was mechanical/non-mechanical (had moving parts or not). 80 images were selected randomly from a set of 512 images depicting everyday objects (Moutsopoulou et al., 2015; Pfeuffer et al., 2017). Sixteen additional images were used in each experiment for a practice run. Items were presented in the center of the screen for 2000 ms, or until response, accompanied by concurrent letter cues on either side of the image indicating the task to apply and the correct response mapping (***Figure 1***). Filler task images were chosen in each block from the remaining unique set (416 images), with the final set size depending on participant response speed. Images in the filler task could be repeated between blocks (on average ~2.5 times). For the size task, the letters ‘S’ (small) and ‘L’ (large) would appear on either side of the item, while the non-mechanical versus mechanical task was cued using ‘N’ (non-mechanical) and ‘M’ (mechanical). The side of the item the letter appeared on indicated the corresponding response button; either the ‘1’ (left) or ‘0’ (right) key on a standard keyboard. Instructing stimulus-response mappings on each trial rather than keeping the mappings constant allowed responses, tasks, and classifications to be orthogonal. After responding, participants were presented with feedback (“correct”, “incorrect”, or “too slow”) for the first 500 ms of a 750 ms inter-trial interval.

The experiment was divided into prime-probe mini-blocks, where each prime-probe mini-block was composed of 16 trials broken down into a prime phase (8 trials) and a probe phase (8 trials), and used a unique set of 8 items. In the original version of the task, these two phases were contiguous, so that each mini-block lasted exactly 16 trials (Whitehead et al., 2020). However, in Experiment 1, we inserted a filler task (of varying duration) between the prime and probe phases of this protocol. Thus, at the end of the prime phase of each mini-block (the first 8 trials), participants were given a short break and instructions for the next section, during which they completed the filler task – a set of identical task-switching trials where novel items that did not repeat were presented – for a set period of time; .5, 1, 3, 5, or 7 minutes. Note that because stimuli were displayed until response (up to 2000 ms), the exact number of trials experienced during these time intervals could vary slightly between participants. After completing the intervening filler task between prime and probe phases, participants were given a self-timed break before continuing on to complete the probe phase (8 more trials) of the prime-probe task. At the end of each mini-block, participants were told their overall accuracy and the length of the remaining task. Participants completed 80 prime and 80 correspondingprobe trials (requiring 80 unique stimuli).

Crucially, whereas we kept the classification task and response mapping constant between the prime and probe trials for each analyzed item (thus controlling for their respective effects), we selectively manipulated whether cognitive control requirements matched (or mismatched) between primes and probes. Specifically, whether a given item occurred on a task repetition trial (same classification task as trial n-1) or on a task switch trial (different classification task from trial n-1) could vary from prime to probe (***Figure 1***). As the first trial of the prime phase of every mini-block could be neither a switch nor repeat trial, and subsequently was not of use to us in the probe stage, we used these items as null trials (i.e., for trial 1) in the probe phase. For these initially presented items, we manipulated the probe classification task, action sequence, and task sequence so as to create a less predictable presentation of the trials of interest (i.e., to prevent the order of image presentation to be repeated between prime and probe sequences). Approximately half of the probes matched their respective primes in terms of control demands (i.e., both were task repeat/switch trials), and half of them were mismatched (i.e., the prime was a task repetition trial but the probe a task switch trial, or vice versa).

### Analysis

As in our previous work (see Whitehead, et al., 2020), analyses were focused on the probe trial response times. In response time analyses, probe trials with incorrect responses were not analyzed. In all analyses probe trials for which the corresponding prime was responded to incorrectly (17.19% error rate, 0.18% non-response rate) and outlier response times (<200 ms and > 2000 ms) were removed prior to analysis. Moreover, items presented as the first prime trial of the mini-block, and corresponding probe trials, were also excluded from analysis. The total time passed between prime and probe presentation was calculated (including the brief breaks) and then trials were sorted into bins according to the five timing groups (i.e. .5, 1, 3, 5, or 7 minutes). We used linear mixed models to analyze these data, instead of a traditional ANOVA, as mixed models are better able to handle unbalanced cell sizes across individuals, which was an issue in our data after the removal of incorrect and slow responses. However, we include a version of the present analysis using an ANOVA design in the supplementary data found on the OSF repository (*https://osf.io/fc3pd/*). Response time data were first submitted to a set of six linear mixed models, in which random effects were modeled identically for all models with an individual participant intercept and an intercept for each image (i.e., a crossed random effect) using the lme4 and lmerTest packages in R (Bates et al., 2015). We had three factors of interest: Prime task sequence (switch vs. repeat – trial n-1 to trial n), Probe task sequence for that same image (switch vs. repeat – trials n-1 to trial n), and Delay (.5 vs. 1 vs. 3 vs. 5 vs. 7 minutes). The hierarchical structure for this set of models can be summarized as Model 1: Null (random effects only), Model 2: Prime task sequence, Model 3: Probe task sequence + Prime task sequence, Model 4: Probe task sequence x Prime task sequence, Model 5: Probe task sequence x Prime task sequence + Delay, Model 6: Probe task sequence x Prime task sequence × Delay. The fit of these mixed models was determined using the anova() command in R to conduct a chi-squared test of each model against its hierarchically subordinate model (i.e., null vs. 1-factor model). We also conducted planned contrasts of the Probe task sequence x Prime task sequence interaction at each delay timepoint in order to fully explore the time-course of stimulus-control binding effects.

### Results

**Figure 2:**
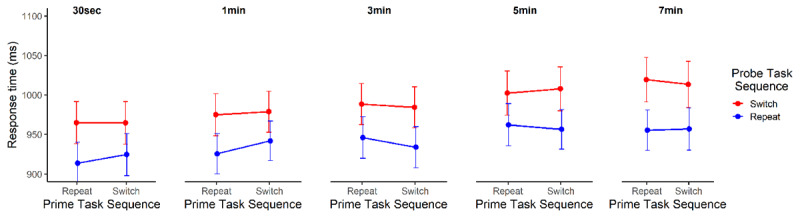
Response times (ms ± 95% estimated confidence intervals) for probe trials, plotted as a function of the Probe Task Sequence (repeat vs. switch), the Prime Task Sequence (repeat vs. switch), and the Delay between prime and probe trials for Experiment 1.

During the delay between prime and probe phases, participants completed 14, 29, 90, 149, and 210 trials on average for each delay length, 0.5, 1, 3, 5, or 7 minutes, respectively (***Table 1***). The results of our primary frequentist mixed model analysis on probe reaction times indicated that model 5, with the inclusion of Probe task sequence and Prime task sequence as main effects and interacting factors, and the inclusion of Delay as a main effect, fit the current data best (p < .001; ***Table 2***). The summary of that model showed that only the main effects of Probe task sequence and Delay were significant (*p*s < .001; ***Table 3***). In addition, the summary of the next hierarchical model, Model 6, which included a three-way interaction between Delay, Probe task sequence, and Prime task sequence did not indicate a significant three-way interaction (*p* = .376). Post-hoc pairwise comparisons of the Probe task sequence × Prime task sequence interaction at each delay length (0.5, 1, 3, 5, or 7 minutes; see ***Figure 2***) indicated no significant interaction effect at any delay level (0.5 minutes: *p* = .747, 1 minute: *p* = .473, 3 minutes: *p* = .534, 5 minutes: *p* = .912, 7 minutes: *p* = .546). Together, this suggests that the formation and implementation of one-shot stimulus-control bindings is not robust to delays between encoding and implementation that involve substantial interference from new, similar event files created from a similar task-switching task.

**Table 1:** Average number of per participant trials submitted to analyses, grouped by fixed factor levels, for Experiments 1, 2, and 3.


			PRIME TASK SWITCH			

			SWITCH		REPEAT	

			PROBE TASK SWITCH			

			SWITCH	REPEAT	SWITCH	REPEAT

Exp 1	Delay (min)	0.5	3.1	2.6	2.8	2.8

		1.0	3.2	2.9	2.9	3.1

		3.0	3.1	2.7	2.8	3.0

		5.0	3.1	2.7	2.7	3.0

		7.0	2.9	2.9	2.6	3.1

Exp 2	Delay (trials)	0–20	14.0	20.2	16.6	17.3

		21–40	14.8	18.3	18.4	15.7

		41–60	14.7	18.5	19.0	15.5

		61–80	15.1	18.1	17.9	15.1

		81–100	12.9	16.6	16.7	13.2

Exp 3	Delay (min)	0.5	3.1	2.6	2.8	2.8

		1.0	2.8	2.6	2.8	2.7

		3.0	3.0	2.7	2.6	2.8

		5.0	2.8	2.6	2.8	2.7


**Table 2:** Results of model comparison for hierarchical models of task-switching for Experiment 1.


		*PARAMETERS*	*AIC*	*logLIK*	*CHI-SQUARED*	*df*	*p*

1.	Null	4	177023	-88507			

2.	Probe Task Sequence	5	176848	-88419	176.83	1	**<.001**

3.	+ Prime Task Sequence	6	176850	-88419	0.23	1	0.630

4.	× Prime Task Sequence	7	176852	-88419	0.10	1	0.755

5.	+ Delay	8	176772	-88378	81.17	1	**<.001**

6.	× Delay	11	176773	-88376	5.00	3	0.172


**Table 3:** Summary results of the Probe task sequence × Prime task sequence + Delay model, in Experiment 1.


	*ß*	*St.Err*	*t*	*p*

Intercept	971.80	10.88	89.36	**<.001**

Probe Task Sequence	–48.58	4.94	–9.84	**<.001**

Prime Task Sequence	–2.78	4.89	–0.57	0.569

Delay	6.41	0.71	9.02	**<.001**

Probe × Prime Task Sequence	2.26	6.95	0.33	0.745


### Discussion

The fact that we did not detect a significant probe task sequence by prime task sequence effect at any of the probed time intervals in Experiment 1 suggests that one-shot stimulus-control bindings in episodic event files are subject to swift decay or overwriting in the face of interference from similar events during the delay between prime and probe sequences. Given that item-specific retrieval effects due to one-shot learning of stimulus-control bindings had previously been observed across a number of experiments with a similar design (Whitehead et al., 2020, 2021), the lack of a stimulus-control binding effect even at short prime-probe intervals seems surprising but is not suggestive of a general lack of replicability. Further, Experiment 1 had other characteristics that distinguished it from prior studies and which may have contributed to these null findings. To foreshadow the results, Experiments 2 and 3 will support the notion that Experiment 1 does not constitute a failure to replicate but points towards the impact of intervening similar events. In particular, Experiment 1 lacked the regular prime-probe structure (involving many recurring stimuli) that had been employed in the previous studies (Whitehead et al., 2020; Whitehead et al., 2021), as the filler task consisted of trial-unique (non-repeating) stimuli. The fact that, overall, there were very few stimulus repetitions in Experiment 1 may have unwittingly disincentivised the encoding and retrieval of these control-level event elements. This aspect distinguishes Experiment 1 from standard prime-probe experiments investigating event file components, where experience with the task over the course of a few trials might lead participants to learn that stimuli recur, which may encourage the encoding and retrieval of event features. Accordingly, in line with previous work showing that event-feature bindings that are not clearly useful to task performance are quite short-lived (Frings et al., 2015), the results of Experiment 1 may thus reflect the fact that participants regarded the encoding and retrieval of item-specific control states as not worthwhile in a setting where only few stimuli ever reappear. In Experiment 2, we therefore sought to again assess the durability of one-shot stimulus-control bindings in the face of intervening events, but this time in a manner that would not disincentivize the formation and retrieval of event files.

## Experiment 2

**Figure 3:**
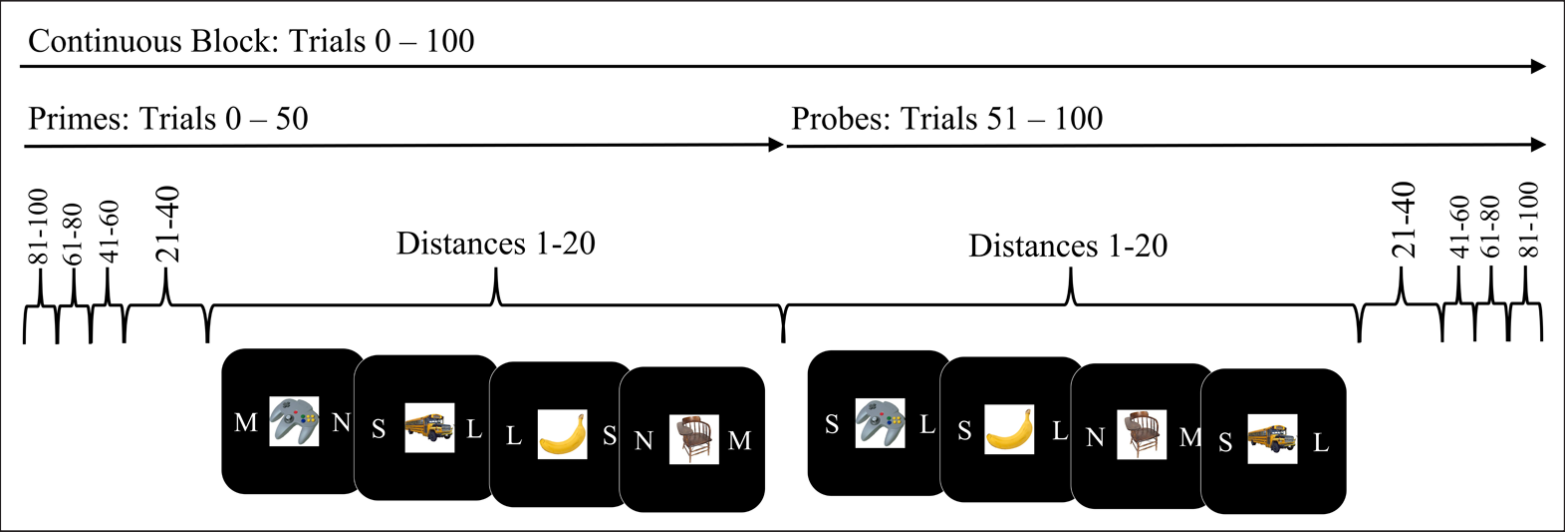
Illustration of the shuffling process for Experiment 2 where primes and probes were submitted to a pseudo-randomized shuffle within 20-trial ‘bins’. The order of presented ‘bins’ during the prime stage was reversed in the probe stage (i.e. if bin order was 1, 2, 3, 4, 5 in the prime, it would be 5, 4, 3, 2, 1 in the probe stage).

Learning strategies can influence the encoding of episodic event files (Giesen & Rothermund, 2015; Giesen et al., 2017, 2020; for review, see Frings et al., 2020), and as discussed above, this may have contributed to the findings of Experiment 1. In Experiment 2, we therefore designed a new varied-duration prime-probe task that aimed to accentuate the usefulness of encoding and retrieving event features compared to Experiment 1. To this end, we presented each stimulus twice, like in traditional prime-probe event coding studies, but here we varied the delay between a prime presentation and probe presentation of each stimulus, from two to 100 trials, in a continuous trial sequence (i.e., a block of 100 trials, see ***Figure 3***). Using this large prime-probe block design allowed us to investigate whether one-shot stimulus-control bindings can be encoded and then implemented when they are clearly task-relevant, but appear without a short, easily recognizable pattern (as in Whitehead et al., 2020). Furthermore, we determined what the durability of these stimulus-control bindings might be in the face of similar, interfering events occurring between prime and probe.

### Method

#### Subjects

For .80 power with an α of .05 to detect item-specific retrieval effects suggesting stimulus-control bindings (i.e., to detect the Prime task sequence X Probe task sequence interaction), using effect size estimates (β = 10) based on previous work (Whitehead, et al., 2020; Brosowsky & Crump, 2018; Pfeuffer et al., 2017), we modeled a set of simulated mixed models using simr() which indicated we needed to run at least 75 participants. Note that the current design required considerably less participants than Experiment 1 due to a large increase in the number of analyzable trials per participant. We recruited 80 participants to complete the task, of which 5 participants were exclude from the final analysis for performing at < 70% accuracy (Whitehead, et al., 2020) resulting in a final sample of N = 75 (mean age = 37.73, SD = 10.65; 35 Women, 40 Men; 63 White). All participants in the current and following experiments were recruited from Amazon Mechanical Turk and provided informed consent in accordance with the policies of the Duke University Institutional Review Board. Participants were compensated at $9 per hour. Workers had to have a US-based IP address, more than 100 approved HITs, and greater than 95% acceptance rate to participate.

#### Procedure

The task-switching task was the same as in Experiment 1 (small/large vs. mechanical/non-mechanical), however, the structure of the task was different. Each participant performed a series of 8 blocks of 100 continuously presented trials, where the first 50 trials were primes and the last 50 trials were probes. Primes and probes were shuffled in such a way as to ‘bin’ distances in pseudo-normal distributions of 20-trial distance increments (see ***Figure 3***). Specifically, images were randomly assigned to one of five bins of 20 images. During probe presentation, item order within each bin was shuffled; however, each bin of images was presented in reverse order as in the prime presentation. For example, if the bin order during the prime phase was [1, 2, 3, 4, 5] then bin order in the probe stage was [5, 4, 3, 2, 1]. Participants completed 800 prime-probe trials (400 prime task sequence trials, and 400 probe task sequence trials), and 16 practice trials before the experiment.

#### Analysis

Data cleaning procedures were identical to Experiment 1. Inaccurate trials were removed (13.10% error rate, 0.08% non-response rate), response times trimmed to be between 200–2000 ms, and the initial trial of each block sequence was removed. Probe trials were analyzed using a series of hierarchical mixed models. Response time data were first submitted to a set of six linear mixed models, in which random effects were modeled identically for all models with an individual participant intercept and an intercept for each image (i.e., a crossed random effect) using the lme4 and lmerTest packages in R (Bates et al., 2015). We had three factors of interest: the Prime task sequence (switch vs. repeat – trial n-1 to trial n), the Probe task sequence for that same image (switch vs. repeat – trials n-1 to trial n), and the distance between prime and probe. Distance was grouped into bins spanning 20-trial distance – i.e., distance 1–20, 21–40, 41–60, 61–80, and 81–100 trials– due to the way it was manipulated experimentally (also in bins of 20-trial distances). The hierarchical structure for this set of models can be summarized as Model 1: Null (random effects only), Model 2: Prime task sequence, Model 3: Prime task sequence + Probe task sequence, Model 4: Prime task sequence × Probe task sequence, Model 5: Prime task sequence × Probe task sequence + Distance, Model 6: Prime task sequence × Probe task sequence × Distance. The fit of these mixed models was determined using the anova() command in R to conduct a chi-squared test of each model against its hierarchically subordinate model (i.e., null vs. 1-factor model). We again completed planned contrasts of the Probe task sequence x Prime task sequence interaction at each delay timepoint in order to fully explore the time course of this interaction, and to match the analysis of Experiment 1.

### Results

**Figure 4:**
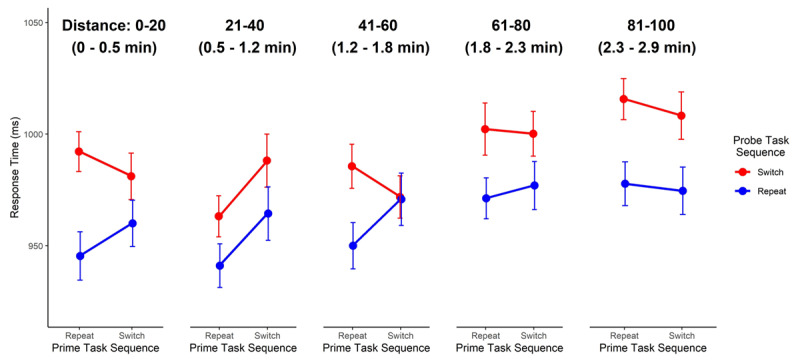
The response times (ms ± 95% confidence intervals) for probe trials, plotted as a function of Probe Task Sequence (repeat vs. switch), Prime Task Sequence (repeat vs. switch), and Distance, the number of trials between the prime and probe presentation of an image, for Experiment 2. Estimated time between prime and probe is displayed below each distance.

The delay between prime and probe phases, for bin distance of 1–20, 21–40, 41–60, 61–80, and 81-100 trials corresponded to an average temporal distance of 23, 69, 114, 161, and 208 seconds (~.5, ~1, ~2, ~3, and ~3.5 minutes), respectively. The results of our primary frequentist mixed model analysis indicated that model 5, with the inclusion of Prime task sequence and Probe task sequence as main effects and interacting factors and the inclusion of Distance as a main effect, fit the current data best (*p* < .001; ***Table 4***). The summary of that model showed that the main effect of Probe task sequence and Distance between prime and probe were significant (*p*s < .001 for each). The interaction between the Probe task sequence and Prime task sequence was also significant (*p* < .011; ***Table 5***). A post-hoc examination of the Probe task sequence x Prime task sequence interaction at each Distance bin length (see ***Figure 4***) from Model 6 (Probe task sequence × Prime task sequence × Distance) indicated a significant interaction effect at distance levels of 1–20 and 41–60 trial (*p* = .037 and *p* = .003, respectively), but not for distances of 21–40, 61–80, and 81–100 between prime and probe (*p* = .851, *p* = .934, *p* = .790, respectively). Together, these results suggest that the formation and implementation of one-shot stimulus-control bindings can be robust to up to 60 intervening trials between encoding and implementation, but given the results of Experiment 1, this durability seems to depend on a task structure where items are regularly repeated.

**Table 4:** Results of model comparison for hierarchical models of task-switching for Experiment 2.


		*PARAMETERS*	*AIC*	*logLIK*	*CHI-SQUARED*	*df*	*p*

1.	Null	4	337365	–168679			

2.	Probe Task Sequence	5	337278	–168634	89.34	1	**<.001**

3.	+ Prime Task Sequence	6	337274	–168631	5.45	1	**0.020**

4.	× Prime Task Sequence	7	337270	–168628	6.06	1	**0.014**

5.	+ Distance	8	337221	–168603	51.00	1	**<.001**

6.	× Distance	11	337224	–168601	3.52	3	0.319


**Table 5:** Summary results of the Probe task sequence × Prime Task Sequence + Distance model in Experiment 2.


	*ß*	*St.Err*	*t*	*p*

Intercept	968.95	23.44	41.34	**<.001**

Probe Task Sequence	–34.43	4.06	–8.48	**<.001**

Prime Task Sequence	–0.41	4.02	–0.10	0.920

Distance	7.33	1.03	7.15	**<.001**

Probe × Prime Task Sequence	14.63	5.77	2.54	**0.011**


### Discussion

These results indicate that the effects of one-shot stimulus-control bindings can be observed after up to 41–60 trials between prime encoding and probe retrieval in the face of interference from similar intervening events (and an average temporal distance of 114 seconds). However, the keen reader will note that while we did see one-shot stimulus-control binding effects at distances 1–20, we did not observe this effect at distances between 21–40. This suggests that under circumstances where stimuli repeat regularly, thus presumably encouraging event file encoding and retrieval, item-specific stimulus-control bindings can be retained and implemented at longer temporal distances than previously seen in Experiment 1. While our post-hoc analysis indicated evidence of one-shot stimulus-control bindings when there were 1–20 or 41–60 trials between prime and probe, for distances of 21–40 or greater than 61 trials between prime and probe, evidence for the implementation of stimulus-control bindings was absent. For the longer distances (> 61 trials), this could be because the temporary stimulus-control bindings had degraded or been overwritten by intervening episodes. However, in the case of shorter distances between prime and probe (21–40 trials), whether there are still intact but inaccessible bindings between stimuli and control states remains a question for future research.

## Experiment 3

While the previous experiments have tested to what degree one-shot stimulus-control bindings are robust to interference from similar events over varying temporal delays, whether these episodic event file bindings are robust to temporal decay alone remains to be tested. In Experiment 3 we therefore tested whether one-shot stimulus-control bindings are robust to temporal decay in the absence of competition from the processing of similar episodes between encoding and retrieval. To this end, we modified the task used in Experiment 1 to instead use an unrelated filler task that did not involve the processing of similar object stimuli during the delay between prime and probe.

### Method

#### Subjects

For .80 power with an α of .05 to detect item-specific retrieval effects suggesting stimulus-control bindings (i.e., to detect the Prime task sequence × Probe task sequence interaction), using effect size estimates (β = |12.5|) based on previous work (Whitehead, et al., 2020; Brosowsky & Crump, 2018; Pfeuffer et al., 2018), we modeled a set of simulated mixed models using simr() which indicated that we needed to run at least 250 participants. We recruited 274 participants to complete the task, of which 31 participants were exclude from the final analysis for performing at < 50% accuracy (Whitehead, et al., 2020) resulting in a final sample of N = 243 (mean age = 37.83, SD = 10.51; 105 Women, 136 Men, and 2 non-responses; 192 White). All participants were recruited from Amazon Mechanical Turk and provided informed consent in accordance with the policies of the Duke University Institutional Review Board. Participants were compensated at $9 per hour. Workers had to have a US-based IP address, more than 100 approved HITs, and greater than 95% acceptance rate to participate.

#### Procedure

The prime-probe task-switching task was the same as in Experiment 1, however, the nature of the intervening task between the prime and probe stage was different. At the end of the prime phase of each mini-block (the first 8 trials), participants were given a short break and instructions for the next section, during which they completed a ‘clock task’ (Lichstein et al., 2000) for a set period of time; 1, 3, 5, or 7 minutes. The ‘clock task’ (see ***Figure 1***) required participants to press the space bar every time an on-screen clock hand rotating clockwise reached the 12 o’clock position. This happened at a random rate between 3 and 10 seconds for the duration of the delay between the prime and probe phases of the task-switching task. After completing the intervening task between prime and probe phases, participants were given a self-timed break before continuing to complete the probe phase of the task-switching task. At the end of each mini-block, participants were told their overall accuracy and the length of the remaining task. Participants completed a total of 128 task switching trials (64 prime and 64 probe trials), corresponding to 8 prime and probe phases of 8 stimuli each. A subset of 64 images were again chosen randomly from a set of 512 images depicting everyday objects (Moutsopoulou et al., 2015; Pfeuffer et al., 2017).

#### Analysis

Data cleaning procedures were identical to Experiment 1, except that trials were sorted into bins according to the four timing groups (i.e. 1, 3, 5, or 7 minutes) based on the total delay between prime and probe presentation (including the brief breaks). In all analyses probe trials for which the corresponding prime was responded to incorrectly (23.84% error rate, 0.07% non-response rate) and outlier response times (< 200 ms and > 2000 ms) were removed prior to analysis. The analysis was identical to Experiment 1 – response time data were submitted to a set of 6 linear models with the same random effects structure. The hierarchical structure for this set of models can be summarized as Model 1: Null (random effects only), Model 2: Prime task sequence, Model 3: Prime task sequence + Probe task sequence, Model 4: Prime task sequence × Probe task sequence, Model 5: Prime × Probe task sequence + Distance, Model 6: Prime task sequence × Probe task sequence × Distance. The fit of these mixed models was determined using the anova() command in R to conduct a chi-squared test of each model against its hierarchically subordinate model (i.e., null vs. 1-factor model). We again completed planned contrasts of the Probe task sequence x Prime task sequence interaction at each delay timepoint in order to fully explore the time-course of this interaction and match the analysis completed in previous experiments.

### Results

**Figure 5:**
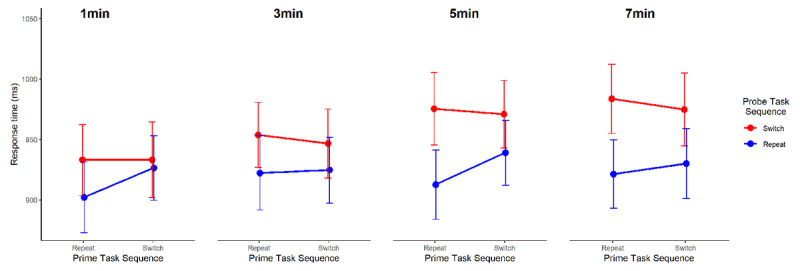
Response times (ms ± 95% estimated confidence intervals) for probe trials, plotted as a function of Probe Task Sequence (repeat vs. switch), Prime Task Sequence (repeat vs. switch), and the Delay between prime and probe trials for Experiment 3.

During the delay between prime and probe phases, participants completed 9, 28, 46, and 60 clock responses on average for each delay length of 1, 3, 5, or 7 minutes, respectively. The results of our primary mixed model analysis indicated that model 5, with the inclusion of Probe task sequence and Prime task sequence as main effects and interacting factors, and the inclusion of Delay as a main effect, fit the current data best (*p* < .001; ***Table 7***). The summary of that model showed that all main effects and interactions were significant (*ps* < .001, except for the main effect of Prime task sequence which was p = .037; ***Table 7***). In addition, the summary of the next hierarchical model, which included a three-way interaction between Probe task sequence x Prime task sequence × Delay did not indicate a significant three-way interaction (p = .376). Post-hoc pairwise comparisons of the Probe task sequence × Prime task sequence interaction against the null at each delay length (1, 3, 5, or 7 minutes) from model 6 (Probe task sequence × Prime task sequence × Delay) indicated a significant interaction effect at delay levels of 1min and 5min (*p* = .016 and *p* = .033, respectively), but not in delays of 3min or 7min (*p* = .095 and *p* = .066, respectively) though the pattern of means was similar. This analysis is visualized in ***Figure 5***. These results suggest that the formation and implementation of one-shot stimulus-control bindings is robust to considerable temporal delays between encoding and implementation.

**Table 6:** Results of model comparison for hierarchical models of task-switching for Experiment 3.


		*PARAMETERS*	*AIC*	*logLIK*	*CHI-SQUARED*	*df*	*p*

1.	Null	4	133654	–66823			

2.	Probe Task Sequence	5	133518	–66754	138.40	1	**<.001**

3.	+ Prime Task Sequence	6	133519	–66753	0.90	1	.344

4.	× Prime Task Sequence	7	133505	–66745	16.02	1	**<.001**

5.	+ Delay	8	133447	–66716	59.37	1	**<.001**

6.	× Delay	11	133446	–66712	6.83	3	.078


**Table 7:** Summary results of the Probe task sequence × Prime Task Sequence + Delay model in Experiment 3.


	*ß*	*St.Err*	*t*	*p*

Intercept	937.42	13.09	301.92	**<.001**

Probe Task Sequence	–26.33	4.87	–5.41	**<.001**

Prime Task Sequence	10.29	4.76	2.16	**0.031**

Delay	5.86	0.76	7.72	**<.001**

Probe × Prime Task Sequence	–27.80	6.81	–4.08	**<.001**


### Discussion

The results of Experiment 3 suggest that one-shot stimulus-control bindings in episodic event files are considerably more durable when there is no interference from similar events between prime and probe (Exp. 3: ~ 5 min; Exp. 2: ~ 2 min). Here, we found evidence of intact one-shot stimulus-control bindings up to 5 minutes after prime presentation (***Figure 5***). For the longest interval, 7 minutes, the lack of evidence for a stimulus-control binding presumably reflects temporal decay. Surprisingly, our planned comparisons again failed to find significant evidence for one-shot stimulus-control associations at the second to shortest interval – here 3 minutes between prime and probe. Whether this is an artifact of the data (e.g., random noise fluctuations) or reflects a cognitive process such as the consolidation of stimulus-control associations in event files – both for Experiment 2 and the present experiment – remains a question for future research. Additionally, future studies investigating stimulus-control bindings at these longer time intervals would be needed to provide corroborating evidence for this interpretation.

## General Discussion

Across three experiments, we sought to delineate the durability of item-specific stimulus-control bindings formed by one-shot learning in a task-switching protocol. We found that retrieval effects of these stimulus-control bindings were observable after up to five minutes between encoding and retrieval (Experiment 3, see ***Figure 5***). This pattern of results was observed under ideal conditions in which there was no intermittent interference from the encoding of similar events between the encoding (prime trial) and retrieval (probe trial) of the corresponding stimulus-control bindings. Under conditions of intermittent interference, we observed a different pattern of results: The durability of one-shot stimulus-control bindings was brief (Experiment 2: ~ 2 min, see ***Figure 4***). Importantly, one-shot stimulus-control bindings were absent when the experimental context disincentivized the use of prior events to inform current or future behavior (Experiment 1, see ***Figures 2*** and ***4***), that is, when the usefulness of encoding and retrieving event files was reduced due to very rare repetition of events (Experiment 1). Taken together, this suggests that episodic event files, specifically stimulus-control bindings, can be quite robust to temporal decay under certain experimental contexts (at least up to the 5 minutes). However, in the face of interference from the encoding of similar events, the ability to encode or retrieve stimulus-control bindings of event files may last a relatively shorter duration (Experiment 2). It should be noted, however, that due to substantial design differences, the result patterns across experiments could not be directly compared.

The current results extend recent work on the encoding and retrieval of episodic event files (Frings et al., 2020; see also Hommel et al., 2001) by considering bindings between stimuli and control states. When placed in the context of prior studies on the durability of bindings between other event file components, it appears that the stimulus-control binding components of event files might be more susceptible to interference from the processing of similar subsequent events than stimulus-action and stimulus-classification bindings formed by one-shot learning (Moutsopoulou et al., 2019; Pfeuffer et al., 2017). The evidence concerning durability of stimulus-response components of event files is somewhat mixed, however, with prior research suggesting that stimulus-classification bindings, as specific type of stimulus-response bindings, can in some cases last over the course of multiple days (Moutsopoulou et al., 2019) but in other cases only several minutes (Pfeuffer et al., 2017) or, in case of different types of stimulus-response bindings, even only seconds (Frings, 2011; Hommel & Colzato, 2004; Moeller, Pfister, Kunde, & Frings, 2016). Moreover, at least for the more short-lived bindings, there is evidence of decay over time (e.g., Hommel & Frings, 2020).

Prior research has demonstrated that the encoding of episodic event files can be influenced by learning strategies (Giesen & Rothermund, 2015; Giesen et al., 2017, 2020; for review, see Frings et al., 2020), and the present study has provided some support that this also applies to the binding of control states to stimuli. In particular, it seems likely that the absence of retrieval effects of stimulus-control bindings observed in Experiment 1 was due to creating an environment where item-specific stimulus-control bindings were rarely useful for efficient task performance, as few stimuli ever reoccurred in that experiment. When there is no clear pattern of repeating events to promote the utility of encoding and retrieving, the potentially burdensome upkeep of item-specific stimulus-control bindings may be disincentivized (e.g., Shenhav et al., 2013) or perhaps the controlled retrieval of these bindings is no longer promoted in such a context (see Hommel, 2022, for corresponding ideas). Conversely, when item-level stimulus-control bindings are incentivized by repeating events – as was the case in Experiments 2 and 3 (as well as in Whitehead et al., 2020, 2021) – they can operate and be retained for a great deal of time (Experiment 3) though this durability is curtailed under conditions of interference from similar events (Experiment 2). Our results show evidence for stimulus-control binding effects, as well as for an effect of a delay/intervening events between the encoding and retrieval of bindings. However, evidence for an interaction between stimulus-control bindings and the delay was equivocal, such that our interpretation of how delay affects stimulus-control bindings is by necessity more speculative. Furthermore, given the long time intervals, by design our experiments were not structured to fully dissociate the contribution of temporal decay in direct comparison to decay as a result of event interference. That is, we could not orthogonally manipulate the delay and the number of intervening events, but rather compared delay conditions with and without intervening similar events. Thus, for longer delays, important gaps in our knowledge on the confounded contributions of time and interference to event file decay remain and we cannot yet fully elucidate the intricate way by which time, events, and contextual task structure may interact in the durability of stimulus-control bindings. Future studies using shorter delay durations that allow for dissociating time and events without introducing unnaturally long waiting periods may further address this question. Nevertheless, we can clearly answer the present study’s research question regarding the maximum durability of stimulus-control bindings: Stimulus-control bindings affect behavior at least up to several minutes. Importantly, this maximum durability is reduced when similar intervening events occur.

Diminishing durability of stimulus-control bindings could be thought of as an inherent limitation. However, a control system attempting to exploit the amalgamation of event file stimulus-control bindings should be flexible enough to incorporate new information to optimize the implementation of control. As such, an interesting question to consider is whether this lack of durability under certain conditions could be a result of strategic modulation at encoding and/or retrieval. On the one hand, one might argue that item-specific effects hinge on the retrieval of stimulus-control bindings (Hommel, 2022). That is, while the encoding of these stimulus-control bindings is automatic, it is the retrieval of these same bindings which is primarily malleable by strategic factors, not the encoding processes (see Whitehead, et al., 2021). Here, the ability to retrieve a single episodic event file might diminish as a function of its relative utility given the current context. On the other hand, the durability of stimulus-control bindings under certain conditions could depend on modulations at encoding. That is, under conditions of many similar events being continuously encoded, weighting the information value of a now-irrelevant stimulus-control binding similar to a new binding could be detrimental to future performance. In either case, our results demonstrate the need for further research on this topic to better understand how stimulus-control bindings are stored in episodic event files, shielded from interference, and then retrieved for implementation. Further, how these bindings might interact with other event file type bindings in episodic memory to facilitate more generalized context-specific implementations of cognitive control, especially in the face of other task-induced regularities, may also be of interest for future studies.

In conclusion, the goal of the present study was to test the durability of one-shot stimulus-control bindings in episodic memory. We found that one-shot stimulus-control bindings are robust to temporal decay for up to at least 5min in the absence of interference from similar intervening events, but that this durability is much reduced (~2 min; and dependent on task statistics) when similar events have to be processed between encoding and retrieval of the association. Thus, interference, rather than temporal decay, may be the main limiting factor on long-term effects of one-shot control learning. These results extend our general understanding of the binding and retrieval of event files in episodic memory (Frings et al., 2020; Hommel et al., 2001) by demonstrating an impact of newly-encoded information on the maintenance and retrieval of previously-encoded bindings. More specifically, these results speak to more recent work on the one-shot binding of stimulus-control states in episodic event files by showing an interfering effect on the maintenance and retrieval of stimulus-control bindings of encoding novel, but overlapping, stimulus-control bindings (Brosowsky & Crump, 2018; Spapé & Hommel, 2008; Whitehead et al., 2020). Future work directed at understanding how contextual regularity of an environment may drive the encoding of stimulus-control bindings or protect against interference from competing events may be fruitful.

## Data Accessibility Statement

Data and code can be found at: *https://osf.io/fc3pd/*.
